# The Dynamic Interactions of m6A Modification and R-Loops: Implications for Genome Stability

**DOI:** 10.3390/epigenomes9020021

**Published:** 2025-06-11

**Authors:** Nicholas Kim, Hong Sun

**Affiliations:** Division of Environmental Medicine, Department of Medicine, New York University Grossman School of Medicine, New York, NY 10010, USA

**Keywords:** R-loops, m6A RNA modification, genome stability, DNA damage

## Abstract

R-loops, three-stranded RNA-DNA hybrid nucleic acid structures, are recognized for their roles in both physiological and pathological processes. Regulation of R-loops is critical for genome stability as disruption of R-loop homeostasis can lead to aberrant gene expression, replication stress, and DNA damage. Recent studies suggest that the RNA modification, N6-methyladenosine (m6A), can modify R-loops and the writers, erasers, and readers of m6A are involved in the dynamic regulation of R-loops. Here, we discuss the reported functions of various m6A regulatory proteins in relation to R-loops, highlighting their distinct roles in recognizing and modulating the formation, stability, and resolution of these structures. We further examine the functional implications of m6A and R-loop interaction in human diseases, with a particular emphasis on their roles in cancer.

## 1. Introduction

R-loops are three-stranded nucleic acid structures formed during transcription when RNA hybridizes with its DNA template, resulting in an RNA-DNA hybrid and an unpaired single-stranded DNA [1,2]. Representing about 5% of the human genome [3], R-loops accumulate in regions such as gene promoters with high GC content [4,5], telomeres [6,7], ribosomal DNA [8], and transcription termination regions [3,9]. R-loops have physiological relevance and play key roles in biological processes including DNA repair, RNA transcription and processing, and gene expression regulation [10]. R-loops at CpG islands (CGIs) help protect these regions from DNA methylation, thereby preventing transcriptional silencing [11]. In addition, R-loops also play a role in immunoglobulin class-switch recombination (CSR) [12]. R-loops assist in the formation of CSR-associated replication origins and enhance the synapsis of recombining switch regions, leading to antibody diversification [12].

R-loops in the genome are regulated through two primary mechanisms: prevention and resolution [1,13]. R-loop accumulation is prevented by coating nascent RNA with processing and export factors during transcription. Topoisomerase I also regulates negative supercoiling behind the transcribing RNA polymerase II, thereby reducing the likelihood of double-stranded DNA opening and forming hybrids [1,8]. Chromatin structure may also play a protective role in that closed chromatin, maintained by histone deacetylation, limits R-loop formation, while open chromatin makes DNA more accessible and prone to hybridization [1,14]. The resolution of R-loops involves the degradation of the RNA strand through the activity of ribonucleases such as RNase H or the unwinding of RNA-DNA hybrids by RNA-DNA helicases [13,15,16]. Disruption of R-loop homeostasis leads to the accumulation of pathological R-loops, which subsequently contribute to aberrant gene expression, replication stress, increased mutagenesis, transcription–replication collisions, DNA DSBs, and telomere instability, ultimately promoting genomic instability [3,17,18,19].

m6A is the most abundant and conserved internal modification in messenger RNA (mRNA) in eukaryotes [20]. m6A is a dynamic and reversible modification regulated by writer proteins such as methyltransferase-like 3 (METTL3), methyltransferase-like 14 (METTL14), and Wilms tumor 1-associated protein (WTAP), as well as eraser proteins like fat mass and obesity-associated (FTO) and AlkB homolog 5 (ALKBH5), and recognized by reader proteins including the YT521-B homology domain-containing family proteins YTHDF and YTHDC, insulin-like growth factor 2 mRNA-binding proteins (IGF2BPs), and heterogenous nuclear ribonucleoproteins (hnRNPs) [21,22,23,24]. m6A plays important roles in various post-transcriptional processes which influence RNA stability, translation, splicing, export, and interactions with RNA-binding proteins [23,25]. m6A modification is involved in numerous biological processes including gene expression regulation, cellular differentiation, and DNA damage response, and thus its dysregulation is involved in the progression of diseases such as cancer [26,27,28].

Recent studies suggest an emerging paradigm in which m6A and R-loops function interdependently, collectively shaping gene expression and genome stability [10,28,29]. While both m6A and R-loops have been independently recognized as essential players in various cellular processes, a growing body of evidence highlights their intricate crosstalk where m6A modification can influence R-loop formation, stability, and resolution [29,30,31,32]. The RNA component of R-loops is subject to m6A deposition by writers such as METTL3 and is recognized by m6A readers like YTHDF2 and YTHDC1, which influence R-loop fate [29,33]. Conversely, disrupting R-loops has been shown to decrease global m6A levels in chromatin-associated RNA (caRNA) [34]. Genome-wide analyses have revealed frequent co-localization between R-loop regions and m6A modification sites, suggesting that R-loops may facilitate the recruitment or activity of the m6A methylation machinery [29]. The interactions between m6A modification and R-loops is a growing area of research, revealing a bidirectional crosstalk that plays a key role in cellular function [10,11,33]. Deciphering the molecular mechanisms underlying this reciprocal influence is crucial for understanding their combined role in genome stability, gene expression, and disease pathogenesis. To further elucidate the significance of this dynamic interplay, this review will focus on the interplay of m6A and R-loops, the biological and biochemical aspects of their interactions, and the relevance of their interplay in the context of human diseases.

## 2. Dynamic Regulation of m6A on R-Loops

The intersection of m6A modification and R-loop biology has emerged as a major regulatory axis in gene expression, RNA metabolism, and genome stability [26,29,32,35]. R-loops are dynamic and subject to various post-transcriptional modifications, including m6A. A growing body of evidence suggests that the m6A modification on R-loops is not a passive mark but actively shapes the structure, stability, and fate of R-loops. Here, we discuss the biochemical basis of m6A deposition, removal and recognition on R-loop structures.

### 2.1. Recruitment of m6A Methyltransferase Complexes to R-Loops: Role of RNA-Binding Proteins

m6A-modified R-loops exhibit a widespread genomic distribution, yet they are preferentially enriched in specific regions. Recent studies employing the coupled use of techniques such as m6A-DNA immunoprecipitation sequencing (DIP-seq) and DNA-RNA immunoprecipitation sequencing (DRIP-seq) have provided insight into the genomic distribution and prevalence of m6A-modified R-loops. Abakir et al. applied this technique in human pluripotent stem cells (hPSCs) to map the distribution of m6A DIP and S9.6 DRIP signals, demonstrating that the majority of RNA-DNA hybrids contain m6A modifications [29]. In hPSC cells, m6A and S9.6 peaks had nearly identical distribution across specific genomic regions and accumulated at transcriptional and repetitive sequences [29]. The co-localization of m6A and R-loops was further confirmed in other mammalian cells including Hela and U2OS cells, as well as plant cells [30,34,36]. While m6A modifications exist on various types of RNA such as mRNA, lncRNA, ribosomal RNA (rRNA), circular RNA (circRNA), and microRNA (miRNA), m6A marks in R-loops are unique as they persist in the RNA strand within RNA-DNA hybrids [29,37]. As methylated adenine takes on a sterically unfavorable conformation to form Watson–Crick base pairing with thymine, m6A located within R-loops may loosen RNA-DNA hybrids, similar to those in RNA duplexes, to permit binding by single-stranded RNA-binding proteins, thus destabilizing R-loops [38,39,40].

A large methyltransferase complex containing METTL3-METTL14 heterodimer catalyzes the transfer of a methyl group from S-adenosylmethionine (SAM) to the N6 position of adenosine residues, primarily within the consensus motif [(G>A)(m6A)C(A/C/U)] (DRACH) [20,24]. In the context of R-loops, METTL3 and METTL14 function as key m6A writers on the RNA component of R-loop structures [41,42,43]. METTL3 is crucial for deposition of m6A on nascent RNAs within R-loop regions, influencing their stability and downstream effects such as transcriptional termination [30]. It is worth noting that, although the DRACH motif is the canonical site for m6A deposition, m6A modification has been identified at non-canonical sequences [20,44], at which the METTL3/METTL14 complex may be recruited via special adaptor proteins.

**Figure 1:**
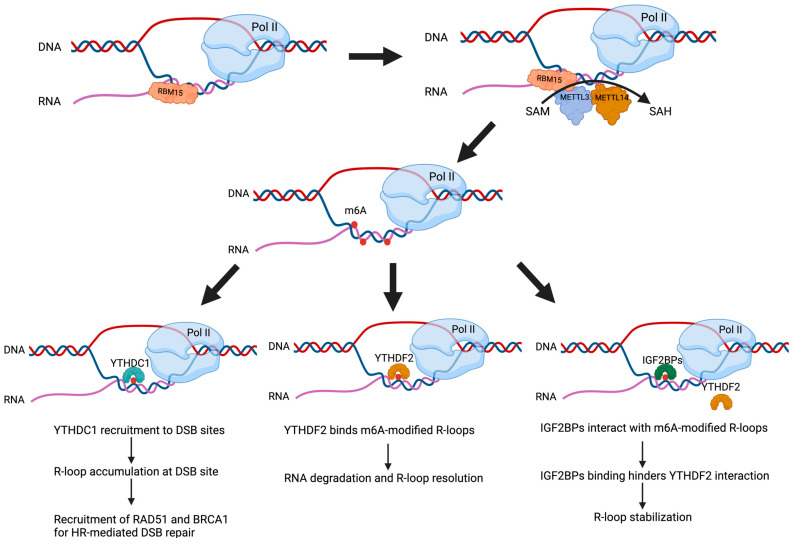
Reciprocal regulation between R-loop dynamics and m6A modification. R-loops can serve as platforms for RNA-binding proteins such as RBM15, which recruit the METTL3-METTL14 m6A methyltransferase complex to deposit m6A marks on nascent RNA within R-loops, using S-adenosylmethionine (SAM) as the methyl donor. The resulting m6A-modified R-loops are recognized by distinct m6A reader proteins such as YTHDC1, YTHDF2, and IGF2BPs. YTHDC1 accumulates at DSB sites, promoting R-loop accumulation and recruiting homologous recombination repair factors such as RAD51 and BRCA1. YTHDF2 facilitates degradation of m6A-marked RNA within R-loops, promoting their resolution. In contrast, IGF2BPs stabilize R-loops by binding m6A-modified RNA and preventing YTHDF2-mediated resolution. Together, these interactions illustrate the dynamic regulation between R-loops and m6A (created with Biorender.com, accessed on 10 June 2025).

The recruitment of m6A machinery to R-loops is facilitated by several RNA-binding proteins. For instance, RNA-binding motif protein 15 (RBM15) has been reported to directly interact with R-loops and promote m6A modification by recruiting METTL3 (as illustrated in Figure 1) [41]. Co-localization studies reveal that endogenous RBM15 overlaps with both R-loops and m6A. Interestingly, RBM15 overexpression enhances the interaction between METTL3 and R-loops, leading to increased m6A deposition, whereas RBM15 depletion results in reduced m6A levels and disrupts the association between R-loops and m6A reader proteins such as IGF2BPs [41]. Similarly, R-loops serve as chromatin anchors for the DEAD-box RNA helicase 21 (DDX21), facilitating the co-transcriptional recruitment of METTL3 [34]. DDX21’s helicase activity is essential for efficient m6A deposition on nascent RNAs by METTL3. Single-stranded DRIP-seq (ssDRIP-seq) revealed a similar enrichment level around both DDX21 and METTL3-caRNA binding sites, suggesting that R-loops play a major role in guiding DDX21 and the MTC to their caRNA targets. Following resolution of R-loops by RNase H treatment, reduced association of DDX21 and METTL3 with target caRNA was observed, underscoring the role of R-loops in anchoring the m6A machinery [34]. In addition to these, transcription factors such as tonicity-responsive enhancer binding protein (TonEBP), which is involved in osmotic regulation and acts as an early sensor of DNA damage, can bind both R-loops and METTL3 through its Rel-homology domain (RHD) [32]. This interaction facilitates METTL3 recruitment to R-loops, particularly at DNA damage sites, leading to enhanced m6A modification [32]. Furthermore, R-loops can recruit the AT-rich interaction domain 1A (ARID1A) protein in an ATM-dependent manner at DNA DSB sites. ARID1A plays a critical role in recruiting both METTL3 and METTL14 to promote the m6A modification of R-loops, which facilitates their resolution and mediates the maintenance of genome stability [45]. Together, these findings highlight a coordinated and dynamic process where R-loop binding proteins target the catalytic m6A methyltransferase complex to R-loops, driving the localized deposition of m6A marks. This process influences R-loop dynamics by modulating their formation, recognition, and resolution, underscoring the interplay between m6A modifications and R-loops in regulating genome stability.

### 2.2. Removal of m6A on R-Loops by FTO and ALKBH5 Demethylases

The removal of m6A is mediated by the demethylases FTO and ALKBH5, members of the AlkB family of non-heme Fe(II)/2-oxoglutarate-dependent dioxygenases [21,22]. Zhang et al. demonstrated that DNA DSBs enhanced m6A modification of R-loops by METTL3 which led to their accumulation. The overexpression of FTO decreased the levels of m6A-modified RNA and R-loops, highlighting the dynamic and bidirectional nature of m6A modification of R-loops [36]. While ALKBH5 and FTO demethylate m6A on RNA and affect numerous aspects of RNA metabolism, more studies are needed to validate whether these demethylases can directly engage with m6A-modified R-loops. In vitro catalytic assays have shown that ALKBH5 can demethylate m6A present on single-stranded RNA [46]. RNA-binding proteins with sequence or structure specificity may also play a role in the recruitment of FTO or ALKBH5 to m6A-modified RNA. For example, the telomeric zinc finger protein ZBTB48 binds U-rich sites near m6A sites on target mRNAs and recruits FTO [47]. Additionally, the RNA helicase DDX46 recruits ALKBH5 to m6A-modified mRNA transcripts through recognition of the conserved CCGGUU sequence [48]. Thus, while ALKBH5 and FTO possess the biochemical machinery to interact with and demethylate m6A, further work is required to elucidate the gap in our standing of whether these erasers can directly interact with m6A-modified R-loops and how m6A demethylation shapes R-loop stability.

### 2.3. Recognition of m6A-Modified R-Loops by Reader Proteins

Functions of m6A modification are delivered by the binding of m6A reader proteins. Various m6A reader proteins have been identified, each playing distinct roles across different aspects of RNA metabolism [49]. The YTH family proteins consist of YTHDF1-3, YTHDC1, and YTHDC2 [49,50]. The YTH domain, a commonly shared domain, has been reported to bind mRNAs containing the conserved G(m6A)C motif [23,50]. For example, using quantitative isothermal titration calorimetry and crystallization techniques, it was revealed that YTHDC1 uses an aromatic cage for m6A recognition and preferentially interacts with the GG(m6A)C sequence [50]. In addition, some proteins without YTH domains displayed a strong capacity binding to m6A modified RNAs, forming the distinct m6A reader families, including HNRNP (HNRNPC, HNRNPG, HNRNPA2B1) and IGF2BP (IGF2BP1-3) families [49]. These proteins possess the unique but highly conserved K-homology (KH) domain that primarily binds to single-stranded RNA and actively participates in RNA metabolism [51]. Unlike YTH domains, which directly recognize m6A marks, KH domains lack a defined binding pocket for m6A marks and thus may interact with m6A-modified local structure [51]. An “m6A switch” model suggests that m6A modification can locally destabilize RNA secondary structure, thereby exposing single-stranded regions that facilitate the binding of RNA-binding proteins such as HNRNPC and IGF2BPs [52]. This model has been further supported by the findings that m6A modifications can induce structural changes in RNA [53].

As indicated earlier, the unique feature of m6A marks on the RNA strand with the R-loop may result in the loosening of the RNA-DNA hybrid, enabling the binding of single-stranded RNA-binding proteins and m6A readers which are crucial for their regulation [33,38,39,40]. The m6A readers YTHDC1, YTHDF1, YTHDF2, HNRNPA2B1, and IGF2BPs have been shown to interact with R-loops [29,36,41]. m6A-modified R-loops accumulate during the S and G2/M phases of the cell cycle in human pluripotent stem cells and are depleted in the G0/G1 phase [29]. YTHDF2 plays a key role in this process by localizing to mitotic chromatin and binding m6A-modified R-loops, particularly in LINE-1 repetitive elements and intronic regions, to facilitate the removal of R-loops during mitosis [29]. This activity is important for preventing the accumulation of co-transcriptional R-loops to maintain genome stability. Thus, YTHDF2-bound R-loops accumulate up to the G2/M phase and are resolved by YTHDF2 during mitosis, highlighting the critical function of YTHDF2 in R-loop homeostasis during cell division. IGF2BPs are also important regulators of m6A-modified R-loops and interact through their four KH domains and two RNA recognition motif domains (RRM) [41,51]. Interestingly, co-immunoprecipitation experiments revealed that IGF2BP proteins interact with each other through their KH domain and may function as a group to stabilize m6A-modified R-loops in gene promoters [41]. IGF2BPs also engage in a competitive interaction with YTHDF2 for m6A-modified R-loop binding (Figure 1) [41]. The YTHDC1 reader can also recognize m6A modifications and play a protective role in R-loops at DSB sites [42]. Collectively, m6A-modified R-loops recruit different m6A readers to fulfill various biological functions.

## 3. The Regulatory Role of m6A in R-Loop Dynamics

m6A modification plays a critical and multifaceted role in the regulation of R-loops, and its dysregulation can significantly contribute to genome instability. While m6A can promote R-loops in certain contexts, it is also important for their proper resolution, and perturbations to these processes may lead to the accumulation of pathological R-loops, increasing the risk of transcription–replication conflicts and DNA DSBs [54,55]. Thus, the balance between m6A writers, readers, and erasers is important in the dynamic regulation of R-loops, and disruptions to this equilibrium can lead to aberrant R-loop accumulation and genome instability.

### 3.1. m6A Promotes R-Loop Formation

Emerging evidence indicates that m6A modification plays a significant role in promoting the formation of R-loops, primarily co-transcriptionally [30,34,36]. Co-transcriptional R-loops promoted by m6A modification have been implicated in slowing down RNA polymerase II and promoting transcriptional termination [30,56]. Promotion of R-loops occurs through various mechanisms involving the activity and recruitment of m6A writers, as well as through the direct effects of m6A on RNA structure and interactions. Various studies have shown that m6A modifications are frequently enriched near the transcription termination sites and this localization is associated with increased R-loop formation in these regions [30,34,36]. Several mechanisms have been presented which suggest that m6A modification of R-loops can promote their formation. For example, the deposition of m6A on nascent RNA by METTL3 was found to promote the formation of R-loops [30,36]. The depletion of METTL3 resulted in decreased R-loop levels, suggesting that m6A modification of the RNA transcript plays a role in facilitating its hybridization with the DNA template [30,57].

m6A modification also has a dynamic role in promoting the formation of R-loops and altering gene transcription [43,58]. Circular RNAs (circRNAs) are single-stranded stable non-coding RNA with a closed, circular structure [59,60]. CircRNAs can function as molecular sponges which bind microRNAs and interact with proteins to regulate gene transcription and are involved in various cancers through regulation of tumor cell invasiveness, migration, and proliferation [59]. m6A modification of chromatin-associated forkhead box protein 1 (FOXP1) was found to facilitate the formation of R-loops between ca-circFOXP1 and the parental gene FOXP1, resulting in transcriptional repression of FOXP1 [58]. Mutation of specific m6A sites on ca-circFOXP1 disrupted R-loop formation and reversed hypoxia-induced proliferation of pulmonary arterial smooth muscle cells [58]. Collectively, these findings highlight the dynamic role of m6A modifications in modulating R-loop formation by facilitating the interaction between circRNAs and their parental genes.

### 3.2. Stabilization of R-Loops by m6A and Reader Protein Binding

In addition to the promotion of R-loop formation, some studies also suggest that m6A modification and machinery are also involved in the stabilization of R-loops. In particular, m6A readers have reported roles in interacting with m6A-modified R-loops and maintaining their stability. The accumulation of m6A-modified RNA through METTL3 has been observed at DSB sites. The reader YTHDC1 is also recruited to DSB sites and prevents the degradation of m6A-modified nascent RNA [36]. YTHDC1 also has a similar protective role through its interaction with m6A-modified TERRA, a long non-coding RNA which can form R-loops with telomeric DNA [61]. TERRA is reported to play an important protective role in the maintenance of telomere stability [61,62]. METTL3-mediated m6A modification of TERRA leads to recognition and stabilization by YTHDC1. In this manner, the writing and reading of m6A modification of TERRA prevents its degradation and promotes the stability of telomeres [61].

IGF2BPs are m6A readers which have RNA recognition motifs and KH domains which allow for recognition and binding of m6A-modified RNA within R-loops [41]. Beyond their function as m6A readers, IGF2BPs have been reported to also function as R-loop readers which stabilize m6A-modified R-loops present within gene promoter regions [41]. An important aspect of their role in R-loop stability is their competitive interaction with another m6A reader, YTHDF2, which is involved in the elimination of m6A-containing RNAs including those present within R-loops (Figure 1). By binding to the same m6A sites on R-loop RNA, IGF2BPs can either displace or prevent YTHDF2 binding and thus enhance the stabilization of R-loops [41].

### 3.3. m6A-Mediated R-Loop Resolution

While m6A modification has an integral role in the formation and stabilization of R-loops, others have also reported that it is critically involved in the resolution of R-loops, preventing excessive accumulation and maintaining genomic stability. The resolution of m6A-modified R-loops is often mediated through interactions with specific m6A readers and the recruitment of enzymes which can dismantle the RNA-DNA hybrid structure [55]. Readers such as YTHDF1, YTHDF2, and HNRNPA2B1 were found to interact with RNA-DNA hybrids [29]. One key player in this resolution process is the m6A reader YTHDF2. The interaction of YTHDF2 with R-loops has been demonstrated by Western blot analysis showing the in vitro binding of YTHDF2 with m6A-modified RNA-DNA hybrid probes [41]. High levels of YTHDF2 co-localization with RNA-DNA hybrids and preferential interaction between YTHDF2 and m6A-containing synthetic RNA-DNA substrates has also been observed [29]. While the depletion of YTHDF2 leads to the accumulation of R-loops, METTL3 knockdown decreased YTHDF2 recruitment to R-loop-containing loci in the genome [29]. Together, these findings suggest that YTHDF2 interacts with m6A-modified R-loops to promote their resolution.

While the mechanisms underlying YTHDF2’s role in R-loop resolution remain unclear, it is thought to aid in the recruitment of enzymes that unwind either RNA-DNA hybrids or nucleases that selectively degrade m6A-modified RNA within these structures [33]. Furthermore, m6A modification functions as a signal for the recruitment of RNase H1, an enzyme which resolves R-loops through the degradation of the RNA strand [45]. Factors such as TonEBP can interact with METTL3, recruiting it to R-loops for m6A modification. Following this, TonEBP recruits RNase H1 to these m6A-modified R-loops to promote R-loop resolution [32]. Similarly, ARID1A also binds to R-loops and recruits METTL3 to DNA DSB sites, increases m6A modification of R-loops, and enhances RNase H1 binding to facilitate R-loop resolution [45]. Interestingly, the impairment of RNase H1 recruitment was observed when catalytically inactive METTL3 was expressed, suggesting that the m6A modification resulting from METTL3 methyltransferase activity is necessary for RNase H1 recruitment and R-loop resolution [45].

Although m6A modification of R-loops can promote their resolution in certain contexts through interactions with RNase H1 and reader proteins such as YTHDF2, it can also regulate R-loop levels by preventing their formation. In glioma stem cells (GSCs), circPOLR2B was discovered to form R-loops with its parental gene, POLR2B, in the nucleus. Elevated levels of YTHDC1 in GSCs enhanced the export of m6A-modified circPOLR2B from the nucleus to the cytoplasm, thereby reducing R-loop formation. This reduction alleviated the suppression of POLR2B transcription and led to increased expression of the PBX1 gene, ultimately promoting malignant behavior in GSCs [43]. Thus, these findings underscore the m6A-mediated regulation of R-loop resolution or prevention through m6A reader interactions.

## 4. Impact of m6A Modification on R-Loops in Genome Stability

R-loops play dual roles in maintaining genome stability. On the one hand, R-loops are involved in many physiological processes such as DNA repair, immunoglobulin CSR [12], gene expression regulation [1], and RNA transcription and processing [10,29]. For example, R-loops contribute to genome stability in part through serving as signaling platforms during DNA damage. They either recruit repair proteins, like Meiotic recombination 11 homolog (MRE11), to DNA double-strand breaks (DSBs), facilitating homologous recombination repair, or stabilize telomeric repeat-containing RNA (TERRA) to maintain telomere-length dynamics [55]. On the other hand, when R-loop homeostasis is disrupted, aberrant R-loop accumulation can promote genome instability by pausing RNA polymerase II or inducing replication fork collision, leading to aberrant gene expression, replication stress, mutations, transcription–replication conflicts, DNA DSBs, and telomere instability [1,18]. For instance, the exposed ssDNA in R-loops is also more vulnerable to damage from nucleases and genotoxins [1]. R-loops contribute to CAG repeat instability through activation-induced deamination followed by base excision repair, increasing DNA breakage and genome instability [63]. R-loops can also be processed into DNA breaks by endonucleases like XPF and XPG during transcription-coupled repair [64]. Thus, the regulation of R-loop dynamics plays a key role in genome stability.

R-loops can form at DNA damage sites and play a regulatory role in the DNA damage response [17,65]. DNA damage, particularly DSBs, has been shown to trigger R-loop formation, which may serve as signaling hubs by anchoring repair factors and stabilizing damaged regions to support the DNA repair process [55]. In response to DNA DSBs, METTL3 was found to be phosphorylated at S43 by ATM and recruited to the damage site, where it catalyzes m6A modification on RNA. This further recruits YTHDC1, promotes the accumulation of RNA-DNA hybrids at DSB sites, and recruits the DNA repair proteins RAD51 and BRCA1 for repair by homologous recombination [36]. Following DNA DSBs, depletion of METTL3 significantly impaired the recruitment of these repair proteins, leading to delayed DNA damage response and reduced repair efficiency [36]. While R-loops play an important role in the DNA damage response, the accumulation of R-loops has also been reported to increase DNA damage in mammalian cells [29]. The interaction of YTHDF2 and m6A-modified RNA-DNA hybrids limits the accumulation of R-loops. The depletion of YTHDF2 led to a marked increase in R-loop accumulation and elevated m6A modifications on RNA-DNA hybrids, indicating that YTHDF2 likely plays a role in processing m6A-modified R-loops. In addition to increased R-loops, YTHDF2 depletion also resulted in the accumulation of γ-H2AX, a marker for DNA DSBs, and reduced cell growth, indicating a role for m6A-mediated R-loop regulation in maintaining genomic stability [29].

Aside from its role in the DNA damage response, the m6A-R-loop axis is also important in the maintenance of telomere stability [61]. m6A modification of TERRA promotes its stabilization and the formation of R-loops through hybridization of m6A-modified TERRA with telomeric repeats. These R-loops are involved in promoting telomeric DNA synthesis and telomeric HR, which has important functions in the DNA damage response to DSBs in telomeres [61,66]. Depletion of METTL3, the major writer involved in TERRA m6A modification, resulted in the shortening of telomeres, accumulation of telomere DNA damage, and an increase in both chromosome ends lacking telomeres and chromosome end-to-end fusions, suggesting that METTL3-mediated m6A modification of TERRA plays an integral role in telomere maintenance [61]. However, while TERRA plays an important protective role in telomeres, it has also been reported that the aberrant upregulation of R-loops results in disruption of the telomere replication fork and DNA damage within telomeres [61,67]. Thus, m6A modification of TERRA is critical in fine-tuning the formation of R-loops to maintain telomere integrity.

Recently, a role for the RNA helicase DDX21 in promoting METTL3-mediated deposition of m6A on R-loops to promote transcription termination and genome stability has been identified [34]. R-loops can function as anchors which facilitate the recruitment of METTL3 by DDX21 in a co-transcriptional manner [34]. Mechanistically, DDX21 unwinds R-loops and allows METTL3 to methylate the nascent RNA, contributing to efficient transcription termination. Thus, the R-loop-DDX21-m6A axis is implicated in the maintenance of genomic integrity, and disruption to this axis can result DNA damage in part by transcriptional readthroughs [34]. Furthermore, depletion of TonEBP, which is involved in the detection and resolution of m6A-modified R-loops through recruitment of RNaseH1, resulted in increased replication stress induced by transcription–replication collision [32]. These findings illustrate the orchestrated interactions between m6A machinery, R-loops, and other proteins which together regulate effective transcription termination and replication stress for maintaining genome stability.

## 5. Functional Implications for m6A and R-Loops in Disease Pathogenesis

The dynamic interplay between m6A modification and R-loops has significant functional consequences for the development and progression of various diseases, particularly cancer and neurological disorders, largely due to its effect on gene expression and genome stability. Genome instability is a key factor in disease pathogenesis associated with the m6A-R-loop axis. Dysregulated R-loop accumulation, often resulting from impaired m6A modification or dysfunctional m6A reader proteins, can obstruct DNA replication and lead to DNA damage [58]. Defects in the proper m6A-mediated resolution of R-loops can also play a role in genome instability [45]. For example, the depletion of METTL3 or TonEBP was found to increase R-loops and decrease cell survival following exposure to DNA damage induced by UV or CPT [32]. Within the context of human disease, dysregulation of m6A and R-loops is implicated in various types of cancers (Table 1).

In PC3 prostate cancer cells, IGF2BP1-3 was found to preferentially interact with m6A-modified R-loops in a KH domain-dependent manner. Additionally, overexpression of IGF2BPs in prostate cancer cells led to an increase in R-loops, suppressed cell migration, and reduced cell growth by upregulating SEMA3F—a gene with tumor suppressor functions in prostate cancer [41]. Mechanistically, IGF2BP-mediated SEMA3F upregulation occurs by hindering DNMT1 binding at SEMA3F promoters. As SEMA3F is correlated with prostate cancer patient survival, the precise regulation of R-loops by m6A and the resulting crosstalk between m6A and DNA methylation play important roles in prostate cancer [41,68,69]. Moreover, RBM15 also mediates m6A modification of R-loops through METTL3 recruitment, and its depletion disrupts the functional activity of IGF2BPs [41].

ARID1A, an SWI/SNF family member, is mutated in nearly 10% of all tumor types such as gastric, endometrial, bladder, and ovarian cancers. Mutations in the ARID1A gene lead to a loss of function which is largely associated with disease progression [70,71]. ARID1A also has a role in the recruitment of METTL3 and METTL14 to R-loops at DSB sites in an ATM-dependent manner to promote HR repair and RNase H1-mediated R-loop resolution [45]. Thus, ARID1A deficiency contributes to R-loop accumulation and promotes tumorigenesis by altering genome stability with defects in HR repair [45,72].

**Table 1:** Genes and proteins involved in R-loop and m6A regulation and associated diseases.

Gene or Protein	Function	Disease	Reference
DDX41	Mutation in RNA helicase hinders YTHDC1 recruitment to R-loops, leading to accumulation of DNA damage and R-loops	Myelodysplastic syndrome	[42]
EWS-FLI1	Promotes R-loop accumulation through enhanced RNA synthesis	Ewing sarcoma	[73]
TERRA	m6A-modified TERRA promotes homologous recombination and protection of telomeres in cancer cells	Neuroblastoma	[61]
ARID1A	Involved in METTL3-m6A axis to enhance RNase-H1-mediated resolution of R-loops	ARID1A altered cancers	[45,70]
SETX	SETX deficiency increased R-loops and DNA DSBs	Ataxia with oculomotor apraxia type 2 (AOA2) and amyotrophic lateral sclerosis type 4 (ALS4)	[74]
circPOLR2B	m6A-modified circPOLR2B interacts with YTHDC1for nuclear transport resulting in reduced R-loop formation in nucleus with parent gene POLR2B	Glioma	[43]
U2AF1, SRSF2, SF3B1	Mutations in splicing factors lead to aberrant accumulation of R-loops	Myelodysplastic syndromes	[75,76]
RBM15	Involved in R-loop recognition and recruitment of METTL3 to R-loops for m6A modification deposition	Prostate cancer	[41]

In myelodysplastic neoplasms, DEAD-box RNA helicase 41 (DDX41) is the most frequently mutated gene [42]. Both R-loop and m6A levels were found to be highly elevated in CD34^+^ cells isolated from MDS patients with DDX41 mutations compared to healthy controls. DDX41 was found to promote YTHDC1 recruitment to R-loops via promotion of METTL3 and YTHDC1 binding, leading to R-loop resolution. This recruitment was hindered in cells with DDX41 deficiency, resulting in increased DNA damage and m6A-modified R-loops [42]. Additionally, the interplay of m6A and R-loops is also involved in the progression of glioma—the most common brain tumor [43]. In glioma stem cells, elevated YTHDC1 expression enhanced the nuclear export of m6A-modified circPOLR2B, reducing the formation of R-loops in the nucleus with the parental POLR2B gene. The decrease in R-loops alleviated transcriptional repression of POL2RB, resulting in increased pre-B-cell leukemia transcription factor 1 (PBX1) expression and promoting malignant cellular behavior [43]. A recent report on pulmonary hypertension has also highlighted the role of m6A in promoting the formation of R-loops between ca-circFOXP1 and host genes, leading to pulmonary vascular remodeling of mouse pulmonary artery smooth muscle cells [58].

Given its important role in tumor formation and progression, there is growing interest in targeting the m6A-R-loop axis for therapeutic intervention. In cancers that rely on the alternative lengthening of telomeres (ALT) pathway, m6A machinery plays a critical role in supporting telomere maintenance [35]. METTL3 and hnRNPA2B1 modify and stabilize TERRA, promoting the formation of R-loops essential for HR-mediated telomere maintenance in ALT-positive cancer cells [35]. Depletion of METTL3 disrupts this process by reducing R-loop formation, impairing RAD51 recruitment, inhibiting telomeric HR, and ultimately causing telomere shortening [6,35]. Small-molecule inhibitors of METTL3, such as STM2457, have shown promise in mouse models of acute myeloid leukemia (AML) and ALT-positive neuroblastoma, highlighting the translational potential for targeting m6A [35,77]. Thus, these findings position METTL3 as an attractive therapeutic target in ALT-positive cancers.

While its role in modulating R-loops appears to be context-dependent, YTHDC1 is also a promising therapeutic target in various cancers. In GSCs, YTHDC1 facilitates the export of m6A-modified circPOLR2B, thus reducing R-loop levels and promoting malignant behavior [43]. Conversely, in ALT-dependent cells, YTHDC1 stabilizes m6A-modified TERRA to enhance R-loop formation and promote telomere stability [61]. A small-molecule inhibitor of histone H3K79 methyltransferase, EPZ-5676, has been reported to inhibit YTHDC1 and impair DNA repair in B-cell lymphoblastic leukemia (B-ALL), resulting in reduced cell proliferation and enhanced cytotoxicity to chemotherapeutics in B-ALL mouse models [78].

Targeting R-loops offers another avenue for therapeutics. While the histone deacetylase (HDAC) inhibitor, Romidepsin, has been shown to inhibit glioblastoma growth in vivo, interestingly, it was also found to promote R-loop formation [43,79]. As the decrease in R-loops in GSCs promoted their malignant behavior [43], Romidepsin may also serve as a potential therapy through its R-loop-promoting function.

In addition to small-molecule inhibitors, CRISPR-Cas13 systems have emerged as powerful tools for RNA-targeted therapies, enabling precise transcriptome editing [80,81]. By fusing catalytically inactive Cas13 (dCas13) with m6A writers or erasers, researchers can control the deposition or removal of m6A modification at specific transcriptomic sites [82,83]. Additionally, abscisic acid (ABA)-based chemically induced proximity (CIP) allows reversible and inducible m6A editing, while photo-caged ABA enables light-controlled m6A modifications [82]. Thus, using CRISPR-Cas13-based technologies hold great therapeutic potential through their targeted manipulation of m6A which could be used in applications such as modifying the stability of tumor suppressor mRNAs [81].

## 6. Conclusions

Although m6A and R-loops have individually been recognized for their critical roles in regulating gene expression, genome stability, and disease pathogenesis, their functional interplay has only recently begun to emerge. This intersection represents a novel and dynamic layer of gene regulation, where m6A marks are deposited, recognized, and removed from RNAs in R-loop structures. This process modulates R-loop formation and resolution, thereby influencing transcriptional activity, genomic integrity, and cellular responses to DNA damage [10,12,31,50].

Despite recent progress, significant gaps remain in our understanding of the m6A-R-loop interplay. While many studies have investigated how m6A modifications regulate R-loop dynamics, the reciprocal effects of R-loops on the activity, specificity, or localization of m6A writers, readers, and erasers are much less understood. Investigating the interaction between m6A and R-loops and identifying the key molecular players involved will be essential for further uncovering the biological impact of their interplay. Utilizing methods such as single-molecule R-loop footprinting (SMRF-seq) for strand-specific mapping of R-loops at near-nucleotide resolution and m6A-SAC-seq for quantitative, single-nucleotide resolution mapping and stoichiometry assessment of m6A may help elucidate the dynamic interplay of R-loops and m6A [84,85]. Additionally, Cas13-based epigenome editing holds great therapeutic potential in targeting the mRNA stability of tumor suppressors or oncogenes. Unraveling the molecular mechanisms governing this interaction will not only enhance our understanding of RNA biology but also open new avenues for therapeutic intervention in diseases characterized by transcriptional and epigenetic dysregulation.

## Data Availability

Not applicable.
